# Images of social support reminders, but not learned safety signals, reduce long-term fear

**DOI:** 10.1038/s41598-025-27689-0

**Published:** 2025-11-28

**Authors:** E. A. Hornstein, M. S. Fanselow, M. G. Craske, Naomi I. Eisenberger

**Affiliations:** 1 Department of Psychology, University of California, Los Angeles, 1285 Franz Hall, Los Angeles, CA 90095 USA; 2 Department of Psychiatry and Biobehavioral Sciences, University of California, Los Angeles, 760 Westwood Plaza, Los Angeles, CA 90095 USA

**Keywords:** Neuroscience, Psychology, Psychology

## Abstract

Although previously considered to simply be a form of safety signals, reminders of social-support-figures have recently been shown to have a distinct combination of effects: inhibiting fear while present, like safety signals do, but also enhancing the extinction of fear, in contrast to safety signals, which impair fear extinction. In this direct comparison of these cues, we confirmed this divergent pattern of effects and revealed that while safety signals inhibit fear by reducing expectation that a fearful event will occur, social support reminders inhibit fear via an alternative pathway, possibly by reducing perceived fearfulness of the event itself. These results substantiate the differentiation of social support reminders from safety signals and contribute to their still-being-discovered effects, paving the way for a possible new understanding of the potential benefits of these reminders for extinction-based therapies. Indeed, while safety signals are harmful to fear extinction processes and the therapies that rely on them, social support reminders may be uniquely beneficial—both decreasing the discomfort associated with completing such therapies by inhibiting fear while present, as well as enhancing therapeutic outcomes by enhancing fear extinction. Thus, reminders of our closest others may represent a simple, inexpensive, and non-invasive method to boost current therapies.

**Supplementary Information:**

The online version contains supplementary material available at 10.1038/s41598-025-27689-0.

## Introduction

Pavlovian fear learning plays a central role in guiding mammalian cognition, emotions, behavior, and, unfortunately, pathology. Dysfunctional fear learning is a hallmark of anxiety disorders, making the excessive or misplaced fears that are characteristic of these disorders extremely difficult to address^1,2^. Although therapies for anxiety disorders capitalize on the learning processes that reduce fear, namely fear extinction, even the most successful therapies—exposure therapies—remain only partially effective. Indeed, not only does drop-out remain an issue, as these procedures are uncomfortable to complete, but weak symptom reduction and relapse remain common even for patients who do complete therapy^1–4^. Thus, methods to boost fear extinction, and consequently augment the success of exposure therapies, have been a main focus of research, leading to numerous breakthroughs^1,2^. Recent work has added to this list of methods to boost fear extinction, demonstrating that the presence of reminders of social support figures during the fear extinction process leads to better and longer lasting reductions in fear^5–7^. Yet, given their recent discovery, the mechanisms underlying these extinction-enhancing effects and their consequent potential to augment exposure therapies remains unclear. Here, we describe new findings that shed light on a novel pathway through which reminders of those we are closest to are able to reduce fear.

The concept that our close others ease fear is not novel. Indeed, a large body of work has demonstrated the powerful ability of social support figures to buffer against psychological and physical stress, perceptions of and responses to threats, and even physical pain, in a process known as social buffering^8–12^. At first glance, the social buffering effects of social support appear to best be described in the same manner as Pavlovian safety signals—reducing expectations of threat by signaling safety from a harmful outcome and ultimately reducing stress and fear^13,14^. Indeed, up until recently, social support figures, and reminders of them, were considered to simply be powerful safety signals. Because of this classification as safety signals, clinicians viewed social support figures and social support reminders as an impediment to long-term fear reduction and treatment for fear disorder. In particular, safety signals, while powerful tools for reducing fear while they are present, have detrimental effects on long-term fear reduction, impairing fear extinction and even preventing it from occurring^13–15^. Thus, if social support reminders are safety signals, they would be expected to be successful at reducing fear *in the moment*, but unsuccessful at reducing fear in the *long-term* (after they are removed) and therefore harmful for extinction-based therapies like exposure therapy.

However, work over the last decade has called this view into question. Findings have revealed that there are two major differences between social support reminders and safety signals. First, while safety signals prevent fear for only the aversive events with which they were trained (e.g., signaling safety from the occurrence of an uncomfortable shock), social support reminders reduce fear for novel aversive events with which they have had no training (indeed, the relationship history with these support figures appears to endow them with the ability to inhibit fear across contexts and types of aversive events^24)^. Second, while social support reminders and safety signals do indeed both reduce fear in the moment^5,13^, they have opposite effects on longer-term fear extinction. Namely, while the presence of safety signals impairs and prevents fear extinction from occurring^15^, reminders of social support figures actually enhance fear extinction and lead to greater fear reduction in the long-term (both directly after their removal and even one day-post fear reduction procedures:^5–7)^. Together, these differences suggest that although they appear to overlap in their in-the-moment fear-reducing effects, social support reminders and safety signals may in fact be engaging diverging fear-reducing pathways that ultimately enable opposite effects on fear extinction processes and long-term fear extinction outcomes.

In order to gain insight into how the fear reduction mechanisms of social support reminders and safety signals might differ, a closer look at safety signals and their effects on fear extinction can be beneficial. Fear extinction is a relatively straightforward procedure during which a cue that is already associated with an aversive outcome (i.e., an image of a blue square that has been learned to signal an accompanying electric shock) is repeatedly presented in the absence of that outcome (i.e., blue square presented without shock), leading to new learning that the cue will not always lead to the expected aversive outcome^14,16^. The success of this procedure relies on an error correction process through which the probability of an expected aversive outcome is updated, such that the greater the initial fear expectation that an outcome will occur, the greater the error correction when that expectation is not met^14,17^. In plain terms, the more you expect something bad to happen, the greater the learning and subsequent reduced fear, when it does not. Thus, fear extinction is more successful when expectation of an aversive outcome is high^14,18,19^.

Safety signals, by their nature, eliminate the fear expectations^13,15^ that fear extinction relies on. These signals have been learned to indicate that an aversive event will not occur in their presence (signaling “safety” from that event:^13)^ and therefore reduce or entirely prevent any expectation of that aversive event that might be triggered by another cue, context, or behavior. For example, if a safety signal (e.g., a yellow circle learned to indicate that no uncomfortable shock will occur), is paired with a fear cue (e.g., a blue square that has been learned to always be accompanied by shock), it will prevent the expectaction of an aversive outcome that that fear cue may produce (e.g., threat of shock). This lack of expectation for the aversive outcome will reduce in-the-moment fear responding to the fear cue, but if this reduction in expectation occurs during fear extinction procedures, it will also prevent any new extinction learning, since no error correction can occur^13,14^. Therefore, when present during fear extinction processes, safety signals lead to decreased fear expectation which has the consequence of leading to weaker fear extinction, such that the same amount, or even greater, fear occurs following their removal, a phenomenon known as “protection from extinction”^15^.

Because reminders of social support figures do not have this "protection from extinction" effect, and in fact appear to have the opposite effect of enhancing fear extinction^5–7^, it seems unlikely that they achieve their fear reduction effects (either in-the-moment, when they are present, or in the long-term, after their removal and when time has passed since fear extinction procedures were carried out) by “signaling safety” (i.e., signaling that an upcoming aversive event is no longer predicted to occur) and reducing fear expectations, as safety signals do. Thus, while safety signals rely on reduced fear expectations to achieve their effects, social support reminders do not. Yet, no work to date has directly compared the effects of social support reminders and learned safety signals during fear learning. Therefore, in the current work we conducted two studies to examine how the presence of learned safety signals and social support reminders during extinction procedures influenced fear expectations and fear extinction outcomes.

## Methods

We conducted two separate studies for this work. Study 1 directly compared the effects of the presence of learned safety signals and social support reminders during fear extinction. Study 2 replicated these procedures with the addition of trial-by-trial reported fear expectation ratings (expectation that shock would occur). Because the procedures for these studies were nearly identical, we review the methods for both studies in combination.

### Participants

Participants were recruited on the University of California, Los Angeles campus. For *Study 1* participants (n_study 1_ = 32, 17 females, m_age_ = 20) were 38% Asian/Pacific Islander, 38% Caucasian, 23% Latino, and 1% Black/African American. For *Study 2* participants (n_study 2_ = 32, 22 females, m_age_=20.25) were 47% Asian/Pacific Islander, 37% Caucasian, and 16% Latino (please see Supplementary Information (SI) for power analyses).

### Pre-screening procedures

Participants were first asked to complete an in-person pre-screening during which they met with an experimenter in the laboratory. During the pre-screening, they were briefed on all study procedures and were asked a short series of questions to ensure that they were over the age of 18, non-pregnant, and had no history of mental health diagnosis and/or were not taking any mental health related medications.

#### Skin Conductance Response (SCR) test

For these studies, fear responding was assessed via changes in Skin Conductance Response (SCR), for which changes in sweat on the surface of the skin is assessed as an indicator of changes in peripheral nervous system activity^20,21^. Thus, during this pre-screening session, participants underwent a *Skin Conductance Response (SCR) Test* to ensure that their physiological stress responses could be detected by the experimental equipment in the following experimental sessions (please see SI for details). Participants for whom SCR could not be detected were not enrolled in the experiment.

#### Informed consent

Participants who were found to be eligible following the eligibility questions and SCR screening were asked to complete the informed consent process with the experimenter during which they were provided with verbal information regarding study procedures and were able to ask any questions they had. They were then asked to read through and complete an informed consent document that reviewed study procedures, presented information on study risks and methods to mitigate them, and provided contact information for the UCLA Institutional Review Board (IRB: which approved and monitored experimental procedures, see Ethics Declaration). Participants were asked to read through and sign this document in compliance with informed consent procedures.

#### Social support figure identification

If participants passed the eligibility questions and SCR screening and completed the informed consent process, they were asked to identify the person “who provides you the most social support on a daily basis” and to rate how much social support that person provides them each day on a scale of 1–10 (mean social support ratings: m_study 1_= 8.59, m_study 2_ = 9.22). Participants were then required to send a digital image of this person to the experimental team before attending the lab for in-person experimental procedures. If participants could not identify a close other who they rated 7 or higher on this scale, they were excluded from participating in the experimental procedures.

## Experimental procedures

### SCR test

In order to confirm that participants’ SCRs were picked up on the day of the experimental procedure, participants completed the SCR test again (as described in the SI). If their responses could not be detected, they were excluded from completing experimental procedures (study 1: 3 participants excluded; study 2: 4 participants excluded).

### Shock calibration procedure

For these studies, mild electric shocks were used to complete safety training and fear conditioning procedures (see below). Thus, at the start of each participant’s participation, the level of shock to be used in the study procedures was calibrated individually to that person to ensure that the shock was extremely uncomfortable, to allow fear learning to occur, but not painful, to avoid unnecessary distress (please see SI for details).

### Safety signal training

**Fig. 1:**
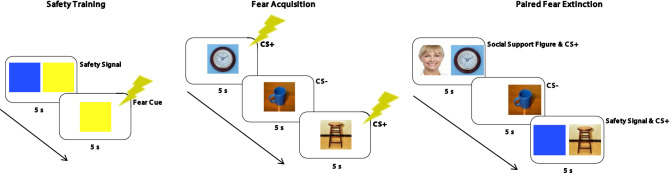
Procedures from *Safety Training*, *Fear Acquisition*, and *Fear Extinction*. Participants were first trained to associate safety from shock with a colored square stimulus (safety training), then were trained to associate fear with two neutral image stimuli (fear acquisition), and then viewed each fear-associated image alongside a safety signal (colored square) or image of social support figure (provided by participant) (paired fear extinction). All ITIs were 10s long, all co-terminating shocks were 200ms, and stimuli were presented for 5s each in a pseudorandom order.

In order for us to examine the effects of safety signals on fear extinction, participants first underwent training to learn to associate safety from the aversive event to be used in the experimental procedures (shock) with a certain cue (the safety signal). Thus, participants viewed two colored square cues (blue, yellow) presented 6 times each. One square was consistently presented alone and paired with a co-terminating 200ms electric shock (fear cue), and the other square (safety signal) was co-presented with the fear cue and no shock occurred (square colors were counterbalanced across participants) (see Fig. 1). All presentations (fear cue alone or safety signal and fear cue pairing) were for 5s each and followed by a 10s inter-trial-interval (ITI) and were pseudorandomized to ensure that no more than 3 trials including shock occurred in a row. Cues during the safety signal training and throughout all other experimental procedures were presented on a PC computer screen using E-Prime 2.0 Professional Software.

Following this procedure, participants provided verbal answers to the experimenter regarding their understanding of which cues were paired with shock and which cues indicated no shock would occur. Along with SCR, these responses were used to assess whether participants were aware of the fear and safety contingencies from the safety training.

### Fear conditioning procedures

Following the safety signal training procedure, participants began the fear conditioning procedures. First, in order to develop fear associations that could later be extinguished, participants underwent a *Fear Acquisition* stage. During fear acquisition, they viewed three neutral object images (clock, stool, cup) presented alone on the screen. Two of the images were consistently paired with a co-terminating 200ms electric shock for 4 presentations each (CS+s: clock, stool) and one image was never paired with shock for 8 presentations (CS-: cup; additional presentations made to ensure that no more than half of the presentations during this stage included shock) (see Fig. 1). This procedure was designed to train participants to associate threat of shock with CS+ images (fear cues). Presentations were pseudorandomized to ensure that no more than 3 shock trials occurred in a row and all image presentations—here and throughout the rest of the fear conditioning procedures—were for 5s followed by a 10s ITI. After this procedure, participants viewed half of a short video clip about airplanes to give them a short break before the next part of the fear conditioning procedure.

Next, participants underwent a *Paired Fear Extinction* stage to examine how fear associations formed during fear acquisition were extinguished. During this stage, each CS+ was consistently presented alongside a secondary image that was either the social support reminder (image of the highly supportive social support figure provided by the participant) or the safety signal (from the safety training procedure), and the CS- was presented alone. No shock occurred on any trial (see Fig. 1). This allowed new learning that the CS+s were not always paired with shock. Pairings of the social support reminder and safety signal were counterbalanced across participants and all presentations were pseudorandomized. After this procedure, participants viewed the second half of the short video clip about airplanes.

**Fig. 2:**
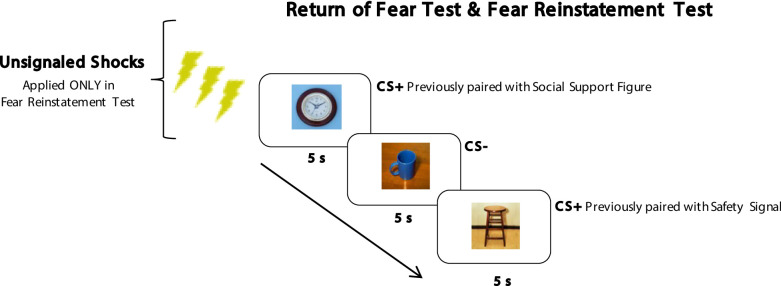
Procedures from *Return of Fear* and *Fear Reinstatment Tests*. All CSs were presented alone, with no paired image and no following shock. Unsignaled shocks were only applied prior to image presentations in the fear reinstatment test (procedures based on^22^. All ITIs were 10s long and all images were presented for 5s each in a pseudorandom order.

After paired fear extinction, participants underwent a *Return of Fear Test* stage to examine the effects of fear extinction. During this test, participants viewed the CS- and each of the CS+s presented alone on the screen for 4 presentations and no shock was administered (see Fig. 2). This allowed us to assess the strength of paired fear extinction as well as any lasting effects of the social support reminder or safety signal pairing. After this stage, participants were sent home.

Finally, participants returned to the lab 24 h later to complete the *Fear Reinstatement Test* stage during which they completed procedures designed to test the strength of fear extinction by fostering the reinstatement of any extinguished fear ^22^. First participants received three unsignaled (no cues were on the screen) 200ms electric shocks followed by 4 presentations each of the CS- and each CS+ presented alone (no shock was paired; see Fig. 2). This provided a second and more stringent test of the lasting strength of fear extinction.

### Fear expectation reporting

In Study 2 only, participants were asked to report on fear expectations by using a dial that moved from 0 to 10. Specifically, they were verbally instructed that each time an image came on the screen, “we would like you to move the dial along the scale from 0–10 to reflect your anticipation of shock on the following scale: where 0 is you 100% *do not* expect to be shocked when this screen occurs and 10 is you 100% *do* expect to be shocked when this screen occurs.” These reports were made on each trial for all stages of the experiment.

### Data analysis strategy

#### Preprocessing

Before analyses were conducted, data were preprocessed according to current recommendations^20,21^ and mean response scores were calculated for each stage to best capture learning (please see SI for details).

So that the effects of safety signals could later be evaluated, we determined whether the presence of the safety signal inhibited fear responding during the safety signal training by evaluating whether the mean response for the safety-signal-and-fear-cue pairing was lower than the fear cue alone. Additionally, so that fear extinction could later be assessed, we determined whether each participant acquired fear during acquisition by assessing whether the acquisition mean for each CS + was higher than the mean for the CS- (CS+ - CS- >0). If fear was not acquired for both CS+s, the participant’s data was excluded from the experiment. If any participant did not show inhibited fear in the presence of the safety signal , their data was excluded from analyses as this indicated that basic safety learning did not occur and the shape did not become a safety signal that could late be used in the safety signal comparison condition.

Using the remaining samples, we ran paired-samples t-tests using means from the safety signal training stage and confirmed that safety signals inhibited fear responding, indicated by significantly lower SCR to the safety-signal-and-fear-cue pairing vs. fear cue alone (study 1: *p* < .001, study 2: *p* < .001, see SI for details). Using means from the fear acquisition stage, we also confirmed that fear was acquired for both CS+s, indicated by significantly higher SCR elicited for each CS+ compared to the CS- (study 1: p’s < 0.001, study 2: p’s < 0.001, see SI for details), and that there was no difference in level of fear responding across CS+s, indicated by no significant difference in SCR across CS+s (study 1: *p* = .999, study 2: *p* = .360).

#### Data analyses

##### Fear responding

For all stages, we first ran a within-subjects ANOVA to assess differences in responding across stimulus types (two CS+s, one CS-), followed by a priori planned, paired-samples post-hoc t-tests to assess fear responding across stimuli.

For the a priori planned paired-samples t-tests, analyses were conducted as follows. First, for each stage the means for each CS+ were compared to the CS-. Second, for each stage, we compared mean difference scores (mean for a CS+ - mean for the CS-) for each condition (social support reminder, safety signal) to examine effects across conditions on fear inhibition (during the paired extinction stage), return-of-fear, and fear reinstatement.

The means from paired fear extinction were used to determine if fear was inhibited in the presence of the social support reminder or safety signal, indicated by no significant difference between a CS+-secondary image pairing and the CS- during the first two trials of this stage.

The means from the return of fear test stage were used to determine if a fear response returned for each CS+ following fear extinction and the removal of the secondary image (social support reminder, safety signal), indicated by significantly higher SCR for a CS+ compared to the CS- during the first two trials of the test stage.

Importantly, as the fear reinstatement test procedure is a test of the strength of fear extinction—it was necessary for some amount of fear extinction to occur during the end of the return of fear test stage, which was the last exposure to the cues before the fear reinstatement stage and represented a second, unpaired extinction procedure. Thus, we also used the final two trials of the return of fear test stage to assess whether fear responses were extinguished, allowing us to assess fear reinstatement 24 h later, indicated by no significant different between a CS+ and the CS-.

Finally, the means from the fear reinstatement stage were used to determine if fear returned in either condition 24 h later and following the fear reinstatement procedure, indicated by significantly higher SCR for a CS+ compared to a CS- during the first two trials of this stage.

##### Fear expectations

For Study 2 only, we examined fear expectation ratings during the paired extinction stage to assess the effect of the presence of a safety signal or social support reminder on expectations of shock when presented with a CS+. To do this, we first averaged across reported expectation of shock during the first two trials of the paired extinction stage for each CS+-secondary image pairing (social support reminder, safety signal) and for the CS-, mirroring the mean scores used to assess SCR. We then compared these mean expectation scores using paired-samples t-tests to examine whether expectations for either CS+-secondary image pairing was significantly higher than for the CS- (which had never been paired with shock and therefore can be used as a baseline), indicating increased expectation of shock.

Although not indicative of how the actual presence of a safety signal or social support reminder influences fear expectations, reported expectation of shock in later stages, when the CS+s were once again presented alone, may offer some insight into expectation effects. Therefore, we averaged across the first two trials of the return of fear and fear reinstatement test stages (once again mirroring the mean scores used to assess SCR) and compared reported expectations for each CS+ (previously paired with safety signal, previously paired with social support reminder) to those for the CS-.

## Results

### Study 1

**Fig. 3:**
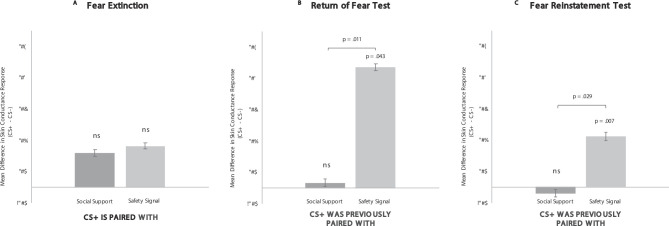
Study 1 Results from *Fear Extinction* & the *Return of Fear and Fear Reinstatement Tests*.

As expected, results from the paired extinction stage revealed no overall interaction in SCR across stimulus types, F(2,62) = 1.18, *p* = .315, η^2^*p* = 0.037, indicating that there were no differences in the fear response that occurred for the CS+ (vs. CS-) paired with the safety signal or paired with the social support reminder; both secondary images reduced fear responding. Post-hoc t-tests confirmed that both conditions inhibited fear by showing that there was no difference between the CS- and either the CS+-safety-signal-pairing or the CS+-social-support-reminder-pairing (p’s > 0.162) (see Fig. 3a). Thus, both social support reminders and safety signals inhibited fear when presented alongside the CS+s.

Results from the return of fear test stage (when the safety signal or social support reminder had been removed) revealed a significant overall interaction across stimulus types, F(2,62) = 4.11, *p* = .021, η^2^*p* = 0.117. Post-hoc t-tests revealed that the CS+ previously paired with the safety signal elicited significantly higher SCR than the CS-, t(31) = 2.11, *p* = .043, 95% CI[0.011,0.602], indicating a fear response occurred during the return of fear test in the safety signal condition, while there was no difference in SCR for the CS+ previously paired with the social support reminder vs.  the CS-, t(31)=-0.581, *p* = .565, 95% CI[-0.364,0.202], indicating no return of fear in the social support condition (see Fig. 3b).Additionally, the CS+ previously paired with the safety signal elicited SCR significantly higher than the CS+ previously paired with the social support reminder, t(31) = 2.70, *p* = .011, 95% CI[0.094,0.679].

Importantly, results from the end of the return of fear test stage revealed that examination of fear reinstatement in the following stage was possible, as the repeated presentations of the CS+s alone and in the absence of shock (unpaired extinction) did lead to some fear extinction by the end of the return of fear test stage (please see SI for details).

Results from the fear reinstatement test stage the next day showed that extinction persisted in the social support reminder condition, but not in the safety signal condition. These results revealed a significant overall interaction across stimulus types, F(2,62) = 4.656, p = .013, η^2^*p* = .131. Post-hoc t-tests revealed that the CS+ previously paired with the safety signal brought about significantly higher SCR than the CS-, t(31) = 2.874, p = .007, 95% CI[.115,.673], indicating the occurrence of a fear response during the fear reinstatement test in the safety signal condition, while the CS+ previously paired with the social support reminder did not(31)=-0.575, *p* = .569, 95% CI[-0.198,0.353], indicating no fear response in the social support condition (see Fig. 3c).Additionally, the CS+ previously paired with the safety signal elicited a significantly higher SCR than the CS+ previously paired with the social support reminder, t(31) = 2.293, *p* = .029, 95% CI[0.035,0.597].

These results confirm expectations. In line with prior learning theory, safety signals led to reduced fear while they were present, but prevented extinction of fear from occurring (protection against extinction:^15^). And, mirroring recent findings, social support reminders both reduced fear while present and led to lasting extinction of fear even after their removal^5–7^. This pattern of effects provides further proof that social support reminders and safety signals are distinct.

### Study 2

**Fig. 4:**
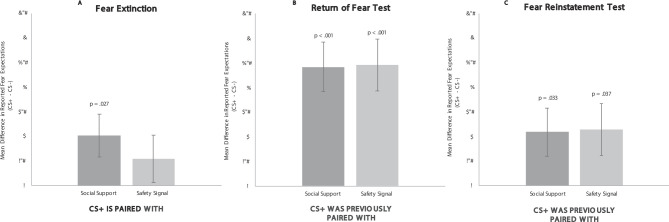
Fear expectation ratings from the *Fear Extinction* & the *Return of Fear and Fear Reinstatement Tests* in Study 2 (reported during first two trials of each stage).

Study 2 replicated these procedures, but also investigated the role of fear expectations. First, examination of the expectation of shock ratings from the first two trials of the paired fear extinction stage (mirroring the trials used to assess fear responding) provided further confirmation that these cues are distinct. Specifically, while expectations when viewing a CS+ paired with a safety signal were no different than those for the CS- (*p* = .261; indicating no shock was expected), expectations when viewing a CS+ paired with the social support reminder were significantly higher than those for the CS-, t(31) = 2.32, *p* = .027, 95% CI[0.061,0.956] (see Fig. 4a). Thus, while there was no expectation of shock in the presence of the safety signal, there was an expectation of shock in the presence of the social support reminder.

When we examined reported expectations of shock in the later return of fear test and fear reinstatement test stages (using the first two trials to once again mirror assessment of fear responding using SCR), we found that, compared to the CS-, expectations were significantly higher for both the CS+ previously paired with the safety signal and the CS+ previously paired with the social support reminder at both the return of fear (p’s > 0.001; Fig. 4b) and fear reinstatement test stages (p’s > 0.05: Fig. 4c) (please see SI for full information and trial-by-trial ratings for all three stages), indicating fear expectations in both conditions. This pattern of effects is expected for safety signals, which should reduce fear expectations while they are present, but lead to rebounding fear expectations after their removal^13,15.^ However, the lasting fear expectations in the social support reminder condition suggests that any later effects of the social support reminder pairing on fear responding (SCR) are not due to lowered fear expectations.

**Fig. 5:**
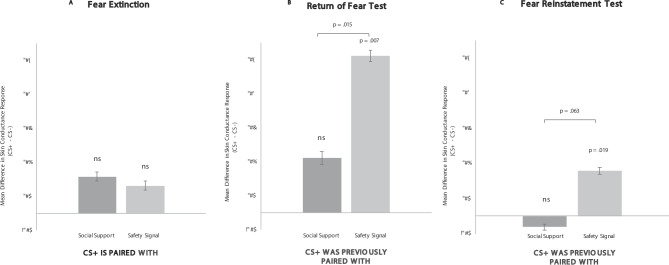
Study 2 Results from *Fear Extinction* & the *Return of Fear and Fear Reinstatement Tests*.

Similar to Study 1, examination of SCR during the paired extinction stage revealed no interaction in SCR across stimulus types, F(2,62) = 1.2451, *p* = .295, η^2^*p* = 0.039 (see Fig. 5a). Post-hoc tests revealed that there was once again no difference in SCR between either CS+ -secondary image pairing (safety signal, social support reminder) and the CS- (p’s > 0.126), indicating fear was inhibited in the presence of both secondary images, just as in *Study 1*.

Results from the next stages followed the same pattern as those from *Study 1*. In particular, results from the return of fear test stage showed a significant overall interaction for SCR across stimulus types F(2,62) = 4.736, *p* = .012, η^2^*p* = 0.133, such that the CS+ previously paired with the safety signal elicited significantly higher SCR than the CS-, t(31) = 2.901, *p* = .007, 95% CI[0.014,0.785], indicating a fear response occurred, while the CS+ previously paired with the social support reminder did not, t(31) = 0.412, *p* = .683, 95% CI[-0.280,0.422], indicating no fear response. Additionally, the CS+ previously paired with the safety signal elicited higher SCR than the CS+ previously paired with the social support reminder, t(31) = 2.562, *p* = .015, 95% CI[0.079,0.701] (see Fig. 5b). Therefore, as in *Study 1*, return of fear occurred for CS+s previously paired with safety signals during extinction, but no return of fear occurred for CS+s previously paired with social support reminders during extinction.

Results from the end of the return of fear test stage showed that it was possible to examine fear reinstatement in both conditions in the next stage, for some amount of extinction occurred when each CS + was repeatedly presented on its own, with no following shock, during this stage.

Finally, results from the fear reinstatement test stage the following day showed that extinction was once again stronger in the social support reminder condition. These results showed a significant overall interaction across stimulus types, F(2,52) = 3.616, *p* = .034, η^2^*p* =  0.122, such that the CS+ previously paired with the safety signal elicited significantly higher SCR than the CS-, t(26) = 2.502, *p* = .019, 95% CI[0.007,0.721], indicating a fear response was reinstated, while the CS+ previously paired with the social support reminder did not, t(26)=-0.633, *p* = .532, 95% CI[-0.210,0.397], indicating no reinstated fear response.Additionally, the CS+ previously paired with the safety signal elicited SCR marginally higher (but nearing significance) compared to the CS+ previously paired with the social support reminder, t(26) = 1.943, *p* = .063, 95% CI[-0.018,0.623] (see Fig. 5c). Thus, the fear response in the social support condition once again did not occur, even after the procedure designed to reinstate it, suggesting stronger extinction occurred in the presence of the social support reminder.

## Discussion

The results of these studies revealed two critical insights into the novel effects of reminders of social support figures on fear reduction. First, they confirmed that social support reminders are distinct from safety signals and do not rely on reduced expectation of an aversive outcome to inhibit fear. Second, they suggested that the abilities of social support reminders to inhibit fear in the moment and enhance fear extinction in the long term might actually rely on separate mechanisms. These new pieces of the puzzle surrounding the recently realized effects of social support during fear learning have implications for future research that could target both scientific understanding of the mechanisms by which social support is able to reduce fear as well the potential of social support reminders to benefit therapeutic application.

Although the previously demonstrated effects of social support reminders during fear learning had hinted that they could not simply be classified as safety signals^23,24^, this direct comparison of the effects of social support reminders and safety signals provides important confirmation. Namely, this work shows that not only do social support reminders not have the same pattern of effects as safety signals, but they also do not rely on reduced fear expectations which are the basis of safety signaling. This confirmation is especially important in light of the fact that previous dismissal of social support reminders, as well as social support figures themselves, in the treatment context was based on the assumption that they engage safety mechanisms, ultimately preventing fear extinction, as safety signals or safety behaviors do. Yet, the theoretical basis from which these assumptions stem relies on the interference of safety-driven reductions in fear expecations during fear extinction processes^13,14^—assumptions which do not hold if social support reminders do not reduce fear expectations. Thus, these results confirm that social support reminders, which had previously been considered to be harmful for fear reduction may in fact be uniquely poised to augment it—both easing the discomfort of fear reduction procedures (by inhibiting fear while present during fear extinction) as well as increasing their effectiveness (by enhancing fear extinction outcomes).

However, if social support is not relying on reduced fear expectations, what pathway are they engaging to inhibit fear? Aside from reduced fear expectations brought about by safety signals, increased reward brought about by reward cues also has an inhibiting effect on fear, yet the pattern of effects of reward cues is also distinct from those of social support reminders^24^. In particular, rewarding cues—cues that signal that a desired event will occur—inhibit fear while they are present (appetitive-aversive interactions:^25,26^), but impair and prevent fear extinction from occuring^27^. Therefore, while rewarding cues share the same short term effects as social support reminders, they diverge in their long-term effects. Similar to safety signals, this pattern of effects (short-term fear inhibition, but no long-term fear extinction) occurs due to alterations in fear expectation. However, while safety signals directly decrease fear expectations, rewarding cues use a less direct path, directly increasing appetitive (reward) expectations, which then counteract (and decrease) fear expectations^14,17,27^. Because this pattern of effects does not align with that of social support reminders, this pathway has been ruled out.

Recent thinking has looked to the social buffering literature to shed light on this issue^24^. Building on the central role of the resources and security provided by close others in enabling social buffering effects and reducing threat and stress, it is possible that social support is not acting on expectations in any way, but is instead acting on assessment of the aversive event itself. Specifically, by increasing perception of available resources and care, social support reminders increase individuals’ perceptions of their ability to cope and recover from potential harm, reducing how threatening or impactful that harm is projected to be. The process by which the value of an expected event is reduced is known as devaluation^28^. An example of devaluation would be a rat that is trained that a cue signals food will be provided, making that cue highly rewarding, but is then overfed so it is no longer hungry, making the food no longer desirable and the cue no longer rewarding even though the food is just as expected. In the case of social support, the aversive event (i.e., shock) may no longer be as threatening because the social support reminder brings to awareness resources and support to cope with any harm that occurs. In plain terms, social support reminders may not be changing how much an event is expected to occur, but how distressing or severe people think the event will be, reducing fear in the moment.

Notably, because this devaluation pathway does not rely on reducing expectations that an event will occur, these expectations remain intact while social support reminders are present during fear extinction—therefore, the error correction processes that drive extinction of fears is able to continue without interruption. This provides a possible explanation as to why social support reminders are able to reduce fear in the moment, like safety signals do, without preventing fear extinction from occurring. Future work examining this devaluation pathway can shed further light on whether the presence of social support reminders might represent a simple, non-invasive method to reduce the discomfort associated with therapies that rely on fear extinction—reducing fear and distress during therapeutic procedures without impairing the processes that bring about long-term symptom-reduction outcomes.

It is important to point out that while results reported here show that social support reminders do not rely on fear expectations to achieve their inbibitory effects on fear in-the-moment, these results do not address the additional ability of social support reminders to then also boost long-term fear reduction via enhanced fear extinction. Though speculative, it is possible that this separate mechanism may rely on the overlapping engagement of the endogenous opioid system for both social connection and fear learning processes. In particular, release of endogenous opioids is triggered in response to close others in order to reinforce and encourage maintenance of social bonds^29,30^ and is separatedly triggered during fear responses to provide analgesia and buffer against expected harm, ultimately providing the negative feedback that drives the error correction system that supports fear association learning^17^. Thus, it is possible that by triggering the release of endogenous opioids, social support reminders lead to additional opioid release that would not normally be included based on fear responding alone, and are consequently able to influence the error correction system and alter long-term fear learning outcomes^24^. However, while much evidence points to this opioid pathway for the long-term fear reduction properties of social support reminders, it has never been directly tested. Therefore, future research should examine the role of social-support-triggered endogenous-opioid-release as a possible pathway for the separate mechanism by which social support reminders are able to enhance fear extinction learning and ultimately lead to greater reductions in fear.

Additionally, it is important to note that, although not directly tested here, the effects of the presence of actual social support figures, not just reminders of them, may require re-examination as well. While safety behaviors (such as seeking out safety cues) have long been considered harmful to fear extinction and treatment procedures^31,32^, safety behavior involving loved ones has never been empirically assessed, but only assumed to interfere. Indeed, some previous work suggests that the presence of actual social support figures during exposure therapies has no detrimental effects^33^ or even provides benefits^34^ to therapeutic outcomes, yet these studies do not directly evaluate safety behavior involving loved ones. These findings, in combination with the confirmation that social support reminders do not belong in the safety signal category, suggest assumptions regarding the presence of social support figures or safety behaviors involving social support figures may require reconsideration as well.

The direct comparison of social support reminders and safety signals provides evidence that these cues are distinct, suggesting that social support reminders facilitate fear extinction without relying on fear expectations. Given that, up until recently, social support reminders were considered to be safety signals, and, because of this classificiation, were considered harmful to fear extinction processes and the therapies that utilize them, this differentiation is critical. Indeed, these results not only cement that social support reminders are a distinct form of fear inhibitors, but also pave the way for a new understanding of the potential of these reminders to benefit extinction-based therapies—reducing the discomfort associated with completing such therapies, by reducing fear while present, while also enhancing therapeutic outcomes, by enhancing fear extinction. Building on these findings, future work must examine whether these effects of reminders of our closest others also occur in those with diagnosed mental health disorder, taking the next step toward understanding whether these reminders represent a simple, inexpensive, and non-invasive method to boost current therapies.

## Supplementary Information

Below is the link to the electronic supplementary material.

Supplementary Material 1

## Data Availability

All deidentified data for this work will be made available on the Harvard Dataverse when this manuscript is in press.
