# Supplementary material for: Images of social support reminders, but not learned safety signals, reduce long-term fear
Source: Sci Rep. 2025 Nov 28;15:43919. doi: 10.1038/s41598-025-27689-0 (PMC12708662; doi:10.1038/s41598-025-27689-0)
Supplement: Supplementary file 1 — Supplementary Material 1 [file 41598_2025_27689_MOESM1_ESM.docx]

Supplementary Information

**Images of social support reminders, but not learned safety signals, reduce long-term fear**

Hornstein, E., Craske., M., Fanselow, M. & Eisenberger, N.

**Methods**

*Power Analysis.*

An a priori power analysis was conducted using data from a similar study in which the impact of added stimuli on fear extinction was examined by evaluating return of conditional fear responses post-extinction (CS+ vs. CS-) (Cohen’s d: .68) (Hornstein, et al., 2016). This analysis revealed that we could evaluate the effects of interest at greater than 95% power (α=.05, two-tailed) using a sample size of n = 30. Based on this analysis which was included and approved in the awarded funding proposal for which this work was conducted (NIH R21MH115287), we collected target samples of n = 30 for each of the studies in the current work and ended with a final sample of n = 32 for each study.

*Participants*.

A total of 72 participants were enrolled for Study 1 and a total of 67 participants were enrolled for Study 2 at the end of the pre-screening session. Participants were then excluded from continuing their participation if they could not select a social support figure who they rated 7 or above on the social support scale during the prescreening session (n _Study 1_ = 1; n _Study 2_ = none) or if they did not pass the SCR test at the beginning of the experimental sessions (n _Study 1_ = 3 participants; n _Study 2_ = 4 participants), and some participants dropped out and did not return for the experimental session (n _Study 1_ =2; n _Study 2_ = 2). After completing their participation, participants were excluded if they were found to be *low responders* (see SCR preprocessing below for more information: n _Study 1_ = 10; n _Study 2_ = 9) or did not acquire a safety association during their safety training test (see Safety Training Check below: n _Study 1_ = 3; n _Study 2_ = 5) or acquire fear associations for both CS+s during the fear acquisition procedure (see Fear Acquisition Check below: n _Study 1_ = 13; n _Study 2_ = 11). Due to technical error (malfunction of SCR data collection equipment or experimenter error in setting up collection equipment), data for some participants was not collected properly and unable to be analyzed (n _Study 1_ = 11; n _Study 2_ = 4).

It is notable that, barring the technical errors, this level of data exclusion is not unusual for similar studies based on the variability of SCR across individuals (SCR test based exclusion), low levels of arousal or attention during fear learning procedures exhibited by some individuals (low responder based exclusion), or difficulty learning for multiple CS+s within one session (fear acquisition based exclusion) (Hornstein, et al., 2016; Hornstein, et al. 2018; Hornstein, et al., 2022; Hornstein, et al., 2024; Olsson, et al., 2005; Schiller, et al., 2010; Phelps, et al., 2004).

*SCR Test.*

Participants’ SCR was measured using the BioPac MP100 system with EDA Isotonic Gel Electrodes, and data were collected using AcqKnowledge 3.9 software (BioPac Systems, Inc., Aero Camino Goleta, CA). For the pre-screening, and at the beginning of all following experimental sessions, electrodes were placed on the fore and middle fingers on the palmar side of participants’ medial phalanges of the left hand. In order to complete the pre-screening, participants were asked to breath in deeply to activate the sympathetic nervous system and to thus increase SCR. If the equipment did not detect a participant’s response, he or she was excluded from the experiment. This same procedure was used in previous work from this team (Hornstein, et al., 2016; Hornstein & Eisenberger, 2017; Hornstein, et al., 2018) and is based on current recommendations (Figner & Murphey, 2011; Lonsdorf, et al., 2017).

*Shock Calibration Procedure.*

At the beginning of each experimental session, participants were set up to with the shock delivery apparatus. Participants received electric shocks via a bar-lead electrode placed on the right wrist. Shock was applied from a SD9 Pulse Stimulator from Grass Technologies (Natus Neurology, Inc.–Grass Products, Middleton, WI).

For the shock calibration procedure (first experimental session only) participants were exposed to a 200ms electric shock starting at 30-volts, and increasing in 5-volt increments. Participants were told the goal was for the shock to reach a level that was “extremely uncomfortable, but not painful” and to inform the experimenter when a level of shock met that instruction. This level of shock was then used for the rest of the experiment. This work-up procedure has been used previously (Hornstein, et al., 2016; Hornstein & Eisenberger, 2017; Hornstein, et al., 2018) and was informed by work-up procedures from prior human fear conditioning studies (Olsson, et al., 2005; Phelps, et al, 2004; Schiller, et al., 2010). For this experiment, average voltage was 55 volts.

**Data Analysis Strategy**

*SCR Preprocessing.*

SCR data were collected and pre-processed using AcqKnowledge 3.9 software. All data were first pre-processed using a low-pass filter and smoothed and were then evaluated using peak-to-peak analysis for each trial during a set response window. In particular, responses were measured as the first peak that occurred between .5s-4.5s after stimulus onset using peak-to-peak amplitude (measured in micro-siemens (μS) (procedures selected based on SCR analysis recommendations; Figner & Murphy, 2011; Lonsdorf, et al., 2017). All measurements were then normalized using a square root transformation and z-scored within each participant using the mean and standard deviation of their SCR responses across all trials in order to retain relative differences in magnitude across learning sessions, while normalizing for individual baseline levels of SCR. The results reported in the manuscript are based on data that have undergone each of these pre-processing steps. To note: because the additional step of calculating z-scores after the square root transformation has not been used in previous published work examining the effects of social support reminders on fear learning (Hornstein, et al., 2016; Hornstein & Eisenberger, 2017; Hornstein et al., 2018; Hornstein, et al., 2022; Hornstein, et al., 2024), we have also included results without the inclusion of z-scores below in the supplemental information (please see the Results section of the SI below). Importantly, the same pattern of effects is found with and without the inclusion of z-scores as shown in the same pattern of effects being reported in the manuscript (based on z-scored data) and in the SI (based on un-z-scored data).

Once measurements were compiled, each trial was evaluated and, based on certain conditions, certain trials were scored as zero response trials or excluded from analysis. A trial was scored as a zero-response trial if there was no peak (no rise in SCR) during the .5-4.5s stimulus window or if no peak amplitude during this window reached .02 μS threshold. Finally, if the participant moved during a trial, as recorded by the experimenter, the trial was excluded from data analysis.

If participants displayed SCR on fewer than 25% of the trials during acquisition, they were considered to be *low responders* and were excluded from analysis. Exclusion criteria described here and above were chosen based on those used in previous human fear conditioning studies (Hornstein, et al., 2016; Hornstein & Eisenberger, 2017; Hornstein, et al., 2018; Olsson, et al., 2005; Schiller, et al., 2010).

*Mean SCR Calculations.*

For the safety training stage SCR was averaged across the final 66% (final 4 of 6 trials) in each condition to capture safety learning and for the acquisition stage, SCR was averaged across the final 75% trials (final 6 of 8 trials CS-; final 3 of 4 trials for each CS+) in each condition to capture fear learning. Based on the different total trial numbers (6 for safety training, 8 (CS-) or 4 (CS+s) for acquisition) these percentages were chosen to be as similar as possible and best capture learning that occurs post initial trials and to model previous research (Hornstein, et al., 2016; Hornstein & Eisenberger, 2017; Hornstein, et al., 2018; Olsson, et al., 2005; Schiller, et al., 2010). For the paired fear extinction stage, SCR was averaged across the first two trials for each CS+-secondary image pairing and the CS- to capture the immediate effect of pairing a social support reminder or safety signal with a CS+ before any new learning has occurred. For the return of fear test stage, we averaged across the first two trials for each CS+ and the CS- to capture the effects of the immediate effects of the paired extinction stage before any new learning (extinction) has occurred and we then averaged across the final two trials of this stage for each CS+ and CS- to examine unpaired fear extinction learning (simple exposure to a CS+ with no ensuing aversive shock). Finally, we averaged SCR across the first two trials of the fear reinstatement test stage to examine whether fear was reinstated immediately following a reinstatement procedure.

*Safety Signal Training Check.*

In order for assessment of the impact of learned safety signals to be conducted, it was first required that each participant acquired a safety association for the safety cue during the safety signal training procedure. Thus, it was evaluated whether each participant’s mean SCR for the safety-signal-and-fear-cue pairing was lower than for the fear cue alone. If it was not, that participant’s data was excluded from further analysis (study 1: 3 excluded; study 2: 5 excluded).

After this, we ran a paired samples t-test comparing mean SCR across trial types and found that in both studies, mean SCR for the safety-signal-and-fear-cue pairing was significantly lower than for the fear cue alone (study 1: t(31)=8.140, p<.001, 95% CI[.77,1.29] ; study 2: t(31)=9.080, p< .001, 95% CI[.91,1.43]), showing that both safety cues acquires safety associations in the analyzed sample.

*Fear Acquisition Check.*

In order for fear extinction to later be assessed, it was also required for participants to acquire fear associations for both CS+s during the fear acquisition stage. Therefore, it was evaluated whether mean SCR for each CS+s was greater than the mean SCR for the CS- for each participant. If it was not, that participant’s data was excluded from further analysis (study 1: 13 excluded; study 2: 11 excluded).

After this, we ran a within-subjects ANOVA to assess differences in responding across the stimulus types (two CS+s, one CS-) using mean SCR from the acquisition stage, followed by a priori planned post-hoc tests. We found that for both studies, there was a significant interaction (study 1: f(2,62)=60.29, p<.001, η^2^p=.660; study 2: f(2,62)=52.87, p<.001, η^2^p=.630) such that in both studies, mean SCR for both CS+s was greater than for the CS- (study 1: t _CS+ 1_(31)=9.83 p<.001, 95% CI[.73,1.11], t _CS+ 2_(31)=8.92, p<.001, 95% CI[.71,1.13]; study 2: t _CS+ 1_(31)=10.64, p<.001, 95% CI[87,1.28], t _CS+ 2_(31)=9.68, p<.001, 95% CI[.75,1.14]) and mean SCR was not difference across the two CS+s (study 1: p=.999; study 2: p=.360).

**Results**

*Results using Pre-processing procedures from previous work (without z-scores)*

Extinction

Results from the paired extinction stage revealed no overall interaction in SCR across stimulus types in study 1 (F(2,62)=1.39, p=.256, η^2^p=.043) or study 2 () and post hoc tests showed no difference between the CS- and either the CS+-safety-signal-pairing or the CS+-social-support-reminder-pairing (study 1: p’s > .116; study 2 p’s >).

Return of Fear Test

Results revealed a significant overall interaction across stimulus types in study 1 (F(2,62)=3.85, p=.027, η^2^p =.110.) and study 2 () and post-hoc t-tests revealed that compared to the CS-, CS+s previously paired with the safety signal elicited significantly higher SCR (study 1: t(31)=2.09, p=.044, 95% CI[.001,.094]; study 2: ), while CS+s previously paired with the social support reminder did not (study 1: t(31)=.622, p=.539, 95% CI[-.062,.033]; study 2: ), and that the safety-signal-paired CS+s elicited SCR significantly higher than the social-support-reminder-paired CS+s (study 1: t(31)=2.56, p=.016, 95% CI[.013,.111]; study 2: ).

Fear Reinstatement Test

Results revealed a significant overall interaction across stimulus types in study 1 (F(2,54)=3.71, p=.031, η^2^p =.121) and study 2 (), and post-hoc t-tests revealed that compaired to the CS- the CS+s previously paired with the safety signal brought about significantly higher SCR (study 1: t(27)=2.09, p=.046, 95% CI[.001,.117]; study 2: ), while the CS+s previously paired with the social support reminder did not (study 1: t(27)=-.228, p=.822, 95% CI[-.059,.047]; study 2: ), and that the safety-signal-paired CS+s elicited a significantly higher SCR than the social-support-reminder-paired CS+s (study 1: t(27)=2.60, p=.015, 95% CI[.014,.116]; study 2: ).

*Study 1.*

Fear Extinction (end of test stage)

Examination of SCR from the final two trials of the test stage revealed that fear extinction did occur for both CS+s. Namely, there was no significant overall interaction (p=.350), showing that there was no significant difference in SCR across the two CS+s and the CS-, and there was no significant difference in SCR for either CS+ compared to the CS- (p’s>.073) and there was no difference in SCR across the two CS+s themselves (p=.664). Based on these results, there was no fear responding occurring for either CS+ at the end of the test stage and fear reinstatement could be examined at the beginning of the next stage.

*Study 2.*

Fear Extinction (end of test stage)

Examination of SCR from the final two trials of the test stage revealed that fear extinction did occur for both CS+s. In particular, there was no significant overall interaction (p=.220) and no significant difference in SCR for either CS+ compared to the CS- (p’s > .264) or across the two CS+s (p=.107). Thus, fear reinstatement could be examined at the beginning of the next stage.

Fear Expectation Ratings

Trial-by-trial ratings (averaged across participants) of how much shock was expected on each trial during the paired extinction stage, return of fear test stage, and fear reinstatement stage, as reported by participants. Ratings were made using a dial connected to AcqKnowledge, with the dial moving from 0-10 (recordings in AcqKnowledge were on a scale from 0-5 and thus multiplied by two to match the scale used by participants.

Trials of interest (first two trials of each stage) are indicated in a red box. The other trials of each stage demonstrate learning of actual shock contingencies (rapid updating of expectations when no shock occurred on any trials in all three stages). This explicit awareness of shock contingencies shows that participants were paying attention.

*

ns

The pattern of effects for the first two trials is replicated when examining reported expectations from the first trial alone, before any learning that no shock would occur had happened—showing that SCR is higher for the CS+ that is paired with the social support reminder vs the CS- (t(31)=2.94, p=.006, 95%CI[.60,3.31], but not for the CS+ that is paired with the safety signal vs the CS- (p=.115).

**

both

Evaluation of mean expectation ratings from the first two trials of the return of fear test stage (mirroring trials used to create mean SCR for that stage) revealed that, compared to the CS-, shock expectation ratings were significantly higher for both the CS+ previously paired with the safety signal (t(31)=4.67, p<.001, 95% CI[1.39,3.54]) and the CS+ previously paired with the social support reminder (t(31)=4.82, p<.001, 95% CI[1.39,3.44]) and that there was no difference in expectation ratings across the CS+s (p=.871). These comparisons were the same when examining reported expectations from the first trial alone, before any learning that no shock would occur had happened—showing SCR was significantly higher for the CS+ previously paired with safety signal vs. CS (t(31)=4.81, p<.001, 95% CI[1.17,2.90]) and the CS+ previously paired with the social support reminder vs. the CS+ (t(31)=4.81, p<.001, 95% CI[1.14,2.83]).

both

*

Evaluation of mean expectation ratings from the first two trials of the fear reinstatement test stage (mirroring trials used to create mean SCR for that stage) revealed that, compared to the CS-, shock expectation ratings were significantly higher for both the CS+ previously paired with the safety signal (t(31)=2.17, p=.037, 95% CI[.071,2.21]) and the CS+ previously paired with the social support reminder (t(31)=2.08, p=.033, 95% CI[.096,2.08]) and that there was no difference in expectation ratings across the CS+s (p=.872). These comparisons were similar when examining reported expectations from the first trial alone—showing SCR was marginally higher for the CS+ previously paired with safety signal vs. CS (t(31)=1.98, p=.057, 95% CI[-.057,3.92]) and the CS+ previously paired with the social support reminder vs. the CS+ (t(31)=2.06, p=.048, 95% CI[.021,3.87]).

**References**

Figner, B., & Murphy, R.O. (2011). Using skin conductance in judgment and decision making research. In M. Schulte-Mecklenbeck, A. Kuehberger, & R. Ranyard (Eds.), *A handbook of process tracing methods for decision research* (pp. 163-184). New York, NY: Psychology Press.

Hornstein, E.A. & Eisenberger, N.I. (2017) Unpacking the buffering effect of social-support figures: Social support attenuates fear acquisition. *PLoS ONE, 12*, e0175891.

Hornstein, E.A., Fanselow, M.S., & Eisenberger, N.I. (2016) A safe haven: Social-support figures as prepared safety stimuli. *Psychological Science, 27,* 1051-1060.

Hornstein, E. A., Haltom, K. E., Shirole, K., & Eisenberger, N. I. (2018). A unique safety signal: Social-support figures enhance rather than protect from fear extinction. Clinical psychological science, 6(3), 407-415.

Hornstein, E. A., Fanselow, M. S., & Eisenberger, N. I. (2022). Warm hands, warm hearts: An investigation of physical warmth as a prepared safety stimulus. *Emotion*, *22*(7), 1517.

Hornstein, E. A., Leschak, C. J., Parrish, M. H., Byrne-Haltom, K. E., Fanselow, M. S., Craske, M. G., & Eisenberger, N. I. (2024). Social support and fear-inhibition: an examination of underlying neural mechanisms. *Social Cognitive and Affective Neuroscience*, *19*(1), nsae002.

Lonsdorf, T. B., Menz, M. M., Andreatta, M., Fullana, M. A., Golkar, A., Haaker, J., Heitland, I., Hermann, A., Kuhn, M., Kruse, O., Drexler, S. M., Meulders, A., Nees, F., Pittig, A., Richter, J., Romer, S., Shiban, Y., Schmitz, A., Straube, B., Vervliet, B., Wendt, J., Baas, J. M. P., & Merz, C. J. (2017). Don’t fear ‘fear conditioning’: Methodological considerations for the design and analysis of studies on human fear acquisition, extinction, and return of fear. *Neuroscience & Biobehavioral Reviews*, *77*, 247-285.

Olsson, A., Ebert, J.P., Banaji, M.R., & Phelps, E.A. (2005). The role of social groups in the persistence of learned fear. *Science, 309*, 785-787.

Phelps, E.A., Delgado, M.R., Nearing, K.I., & LeDoux, J.E. (2004). Extinction learning in humans: Role of the Amygdala and vmPFC. *Neuron, 43*, 897-905.

Schiller, D., Monfils, M., Raio, C.M., Johnson, D.C., LeDoux, J.E., & Phelps, E.A. (2010), Preventing the return of fear in humans using reconsolidation update mechanisms. *Nature, 463,* 49-53.
